# A Novel Gemcitabine-Resistant Gallbladder Cancer Model Provides Insights into Molecular Changes Occurring during Acquired Resistance

**DOI:** 10.3390/ijms24087238

**Published:** 2023-04-14

**Authors:** Luis Vergara-Gómez, Carolina Bizama, Jun Zhong, Kurt Buchegger, Felipe Suárez, Lorena Rosa, Carmen Ili, Helga Weber, Javiera Obreque, Karena Espinoza, Gabriela Repetto, Juan C. Roa, Pamela Leal, Patricia García

**Affiliations:** 1 Biomedicine and Translational Research Laboratory, Centre of Excellence in Translational Medicine and Scientific and Technological Bioresource Nucleus (CEMT-BIOREN), Universidad de La Frontera, Temuco 4810296, Chile; luisosvaldo.vergara@ufrontera.cl (L.V.-G.);; 2 School of Medicine, Department of Pathology, Pontificia Universidad Católica de Chile, Santiago 8330024, Chile; cbizamas@uc.cl (C.B.);; 3 Center for Cancer Prevention and Control (CECAN), Pontificia Universidad Católica de Chile, Santiago 8331150, Chile; 4 Delta Omics Biotechnology, Rockville, MD 20855, USA; 5 Department of Basic Sciences, Universidad de La Frontera, Temuco 4810296, Chile; 6 Laboratory of Integrative Biology (LIBi), Centre of Excellence in Translational Medicine and Scientific and Technological Bioresource Nucleus (CEMT-BIOREN), Universidad de La Frontera, Temuco 4810296, Chile; 7 Center for Genetics and Genomics, Facultad de Medicina, Clínica Alemana, Universidad del Desarrollo, Santiago 7610658, Chile; 8 Millennium Institute on Immunology and Immunotherapy (IMII), Pontificia Universidad Católica de Chile, Santiago 8331150, Chile; 9 Department of Agricultural Sciences and Natural Resources, Faculty of Agricultural and Forestry Science, Universidad de La Frontera, Temuco 4810296, Chile

**Keywords:** gallbladder cancer, acquired drug resistance, gemcitabine, epithelial-to-mesenchymal transition, gene expression profiling, SILAC-based phosphoproteomics, cytidine deaminase

## Abstract

Treatment options for advanced gallbladder cancer (GBC) are scarce and usually rely on cytotoxic chemotherapy, but the effectiveness of any regimen is limited and recurrence rates are high. Here, we investigated the molecular mechanisms of acquired resistance in GBC through the development and characterization of two gemcitabine-resistant GBC cell sublines (NOZ GemR and TGBC1 GemR). Morphological changes, cross-resistance, and migratory/invasive capabilities were evaluated. Then, microarray-based transcriptome profiling and quantitative SILAC-based phosphotyrosine proteomic analyses were performed to identify biological processes and signaling pathways dysregulated in gemcitabine-resistant GBC cells. The transcriptome profiling of parental and gemcitabine-resistant cells revealed the dysregulation of protein-coding genes that promote the enrichment of biological processes such as epithelial-to-mesenchymal transition and drug metabolism. On the other hand, the phosphoproteomics analysis of NOZ GemR identified aberrantly dysregulated signaling pathways in resistant cells as well as active kinases, such as ABL1, PDGFRA, and LYN, which could be novel therapeutic targets in GBC. Accordingly, NOZ GemR showed increased sensitivity toward the multikinase inhibitor dasatinib compared to parental cells. Our study describes transcriptome changes and altered signaling pathways occurring in gemcitabine-resistant GBC cells, which greatly expands our understanding of the underlying mechanisms of acquired drug resistance in GBC.

## 1. Introduction

Gallbladder cancer (GBC) is the most common neoplasm of the biliary tract, characterized by a marked variation in overall incidence and mortality among different populations. The prognosis for GBC is poor because most cases continue to be diagnosed at advanced stages when complete surgical resection, the only potentially curative treatment, is not possible [1].

Adjuvant systemic therapy is prescribed for most patients after gallbladder resection to decrease the risk of recurrence by eliminating clinically undetectable micrometastatic disease [2]. Nevertheless, recurrence is high and the reported 5-year survival rates are less than 50% for this setting [3,4,5]. For patients with unresectable locally advanced and metastatic GBC, systemic chemotherapy with gemcitabine alone or combined with a platinum compound represents the mainstay of first-line treatment [6,7], whereas combination chemotherapy with 5-fluorouracil (5-FU) plus leucovorin and oxaliplatin (FOLFOX) or 5-FU plus leucovorin and irinotecan (FOLFIRI) are used as second-line regimens [1]. Unfortunately, the effectiveness of any one of these regimens is limited and the median overall survival is less than 1 year [8]. A recent systematic review and meta-analysis showed that radiological response rates for GBC seem to be higher than for other forms of biliary tract cancer, but that does not result in longer progression-free survival (PFS) or overall survival (OS) [9].

The high recurrence rates and limited clinical responses to the current therapeutic regimens reflect the aggressive nature and highly drug-resistant phenotype of GBC cells. The cellular and molecular mechanisms associated with chemoresistance in GBC have been scarcely explored, and most studies have essentially evaluated intracellular mechanisms involved in drug resistance in other cancers, such as the activation of drug transporters [10], the expression of specific microRNAs [11,12], the inactivation of drug-metabolizing enzymes [13], or the presence of cancer stem cells [14].

Gemcitabine is the major cytotoxic agent in the adjuvant setting and standard first-line chemotherapy in GBC, used alone or in combination with other cytotoxic agents. Therefore, understanding the mechanisms and molecular features associated with the development of gemcitabine resistance in GBC is crucial for the development of novel therapeutic strategies aimed at either enhancing its cytotoxic effect or selectively targeting vulnerabilities of drug-resistant tumor cells.

In this study, we investigated the cellular and molecular changes associated with the acquisition of gemcitabine resistance in gallbladder cancer by developing and characterizing two gemcitabine-resistant cell lines (NOZ GemR and TGBC1 GemR). Comparative transcriptome and quantitative phosphotyrosine (pY) proteomic analyses revealed potential mechanisms underlying gemcitabine resistance, including the dysregulation of drug metabolism, the enrichment of the epithelial-to-mesenchymal transition (EMT) process, and the activation of receptor and nonreceptor tyrosine kinases such as ABL1, PDGFRA, and LYN. Moreover, pharmacological inhibition using the multikinase inhibitor dasatinib suppressed the proliferation and migration of NOZ GemR cells, suggesting novel therapeutic targets to overcome gemcitabine resistance in gallbladder cancer.

## 2. Results

### 2.1. Establishment and Biological Characterization of Gemcitabine-Resistant Gallbladder Cancer Sublines

**Figure 1:**
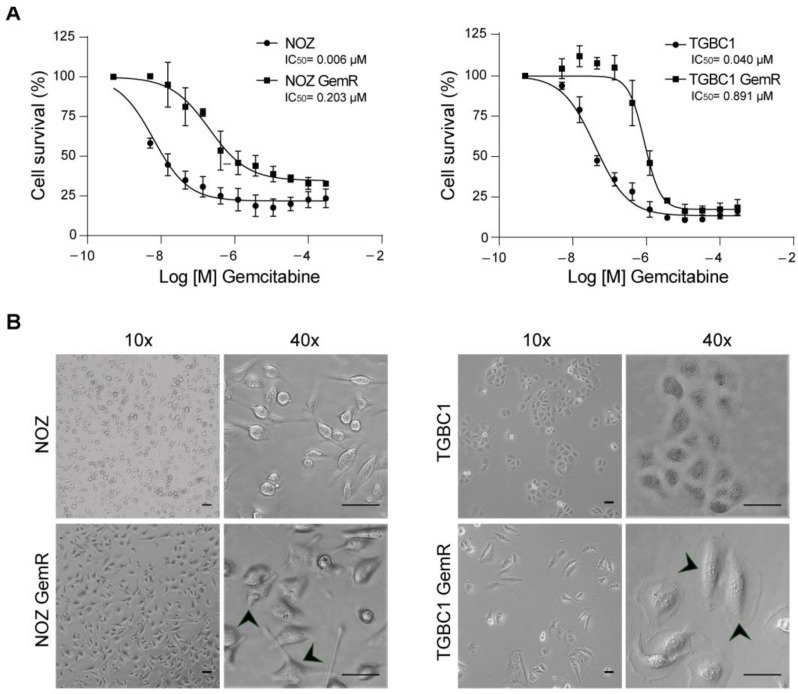
Distinctive features of gemcitabine-resistant gallbladder cancer cells. (**A**) Dose-response curves of gemcitabine-resistant and parental cells after 72 h of incubation with gemcitabine (mean ± SD from three independent experiments with three technical replicates); and (**B**) bright-field images showing morphological characteristics of parental and gemcitabine-resistant cells, and the fusiform shape of resistant cells (arrowheads). Scale bars of 50 µm.

The GBC cell lines NOZ and TGBC1 were used to explore the mechanisms underlying acquired resistance to gemcitabine in GBC. The stable gemcitabine-resistant sublines (NOZ GemR and TGBC1 GemR) showed an increased IC_50_ compared to that of the parental cell lines, with a resistance index (RI) of 32.8 and 22.2, respectively (Figure 1A and Table S1). The gemcitabine-resistant cells also displayed a reduced growth rate (Figure S1), with a significant increase in times of 36.26 and 33.03 h for NOZ GemR and TGBC1 GemR compared to 28.97 and 26.08 h for parental NOZ and TGBC1 cells, respectively.

Regarding morphology, we observed differences in size and shape. Notably, most gemcitabine-resistant cells exhibited an elongated shape, a characteristic change in EMT, and TGBC1 GemR cells were larger and did not grow in clusters like parental TGBC1 cells (Figure 1B).

The acquisition of resistance induced by a drug treatment commonly results in the development of multidrug resistance, which determines cross-resistance to other chemotherapeutic agents with overlapping working mechanisms or even to structurally and functionally unrelated drugs [15]. Since gemcitabine is often administered in combination with cisplatin and sequential second-line systemic therapy for GBC includes 5-FU-based combination chemotherapy [1], we decided to evaluate whether gemcitabine resistance conferred cross-resistance to 5-FU and cisplatin in our in vitro model of acquired drug resistance. The viability assay demonstrated that NOZ GemR cells displayed similar resistance to cisplatin and 5-FU compared to parental cells, exhibiting a slight increase in the IC_50_ values (Table S1). Instead, cross-resistance to cisplatin (RI = 2.9) and 5-FU (RI = 23.6) was observed in TGBC1 GemR (Table S1).

### 2.2. Gemcitabine-Resistant Cells Exhibit an EMT Signature and Dysregulation of Metabolic Pathways

The gene expression profiling revealed a very clear distinction between parental and gemcitabine-resistant cells (Figure S2A). The extensive analysis of GeneChip data showed differential expression of 562 protein-coding genes (DEGs) in NOZ GemR cells, from which 263 were upregulated and 299 were downregulated compared to NOZ parental cells (Figure S2B, Table S2). In TGBC1, from a total of 773 protein-coding DEGs, 280 were upregulated and 493 were downregulated in gemcitabine-resistant cells compared to parental cells (Figure S2B, Table S2).

**Figure 2:**
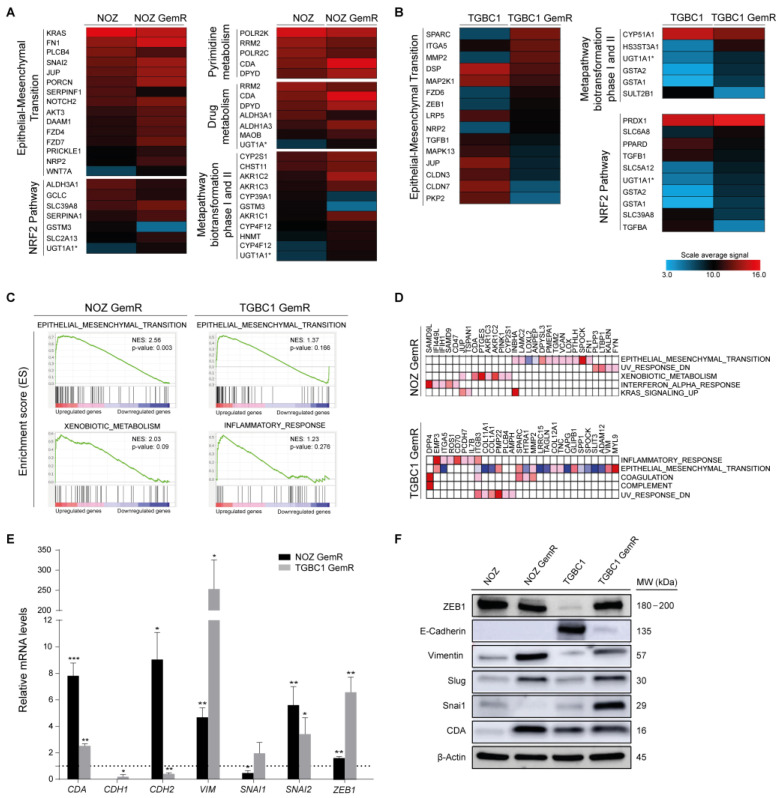
Biological processes dysregulated in gemcitabine-resistant cells. (**A**,**B**) Heat map representation of dysregulated pathways in NOZ GemR and TGBC1 GemR cells compared to parental cells. Data are expressed as the average signal expression of biological triplicates; (**C**) GSEA enrichment plots showing positively enriched biological pathways in each gemcitabine-resistant subline; (**D**) heat maps showing the clusters of upregulated genes (columns) of enriched biological processes (rows) in NOZ GemR and TGBC1 GemR cells according to the leading-edge analysis. The expression values are represented by colors from high (red) to low (dark blue); and (**E**,**F**) gene and protein expression of CDA and EMT regulators. Relative gene expression of each drug-resistant subline is compared to their respective parental cells normalized to 1 (dashed horizontal line). All data are representative of at least three independent experiments (mean ± SD). Statistical significance was determined using a two-tailed Student’s *t*-test with Welch’s correction (* *p* < 0.05, ** *p* < 0.01, and *** *p* < 0.001). Graph bars without an asterisk represent results without statistical significance.

Only 16 upregulated DEGs and 9 downregulated DEGs were shared between NOZ GemR and TGBC1 GemR cells (Figure S2C). However, gene ontology analysis identified several dysregulated biological processes shared by both gemcitabine-resistant sublines, including flavonoid glucuronidation (GO: 0052696), xenobiotic glucuronidation (GO: 0052697), biphenyl catabolic process (GO: 0070980), drug metabolism (KEGG: 00982/00983), and pentose and glucuronate interconversions (KEGG: 00040), among others (Figure S2D). These biological processes are represented by gene families such as UGT, GST, AKR, and ALDH that encode enzymes involved in the metabolism of anticancer agents (Table S3). Consistently, the pathway-driven analysis revealed sets of DEGs involved in the same metabolic pathways as well as in the EMT process (Figure 2A,B). To identify enriched biological processes in the gemcitabine-resistant sublines, we also performed a GSEA analysis by examining the DEGs ranked according to their expression level. The results confirmed that the epithelial–mesenchymal transition hallmark was significantly enriched in NOZ GemR cells (NES: 2.56; *p* = 0.003), along with the enrichment of other biological processes in both GemR cells (Figure 2C, Table S4). The enrichment of each hallmark is promoted by a subset of overrepresented DEGs, called core genes [16], which could play a key role in the chemoresistant phenotype of GemR cells. Based on the leading-edge analysis, we identified core genes that overlapped among the top five most positively enriched gene sets (Figure 2D, Table S5). Many of them have been associated with the acquisition of malignant properties and/or chemoresistance in other tumors, including *DPP4*, *ITGB3*, and *SPARC* (identified in TGBC1 GemR cells), as well as *FN1*, *LAMC2*, *PTGES*, *FYN*, and *CDA* (identified in NOZ GemR cells).

According to the transcriptomics data, the EMT process was particularly enriched in both gemcitabine-resistant sublines, and further validation by qRT-PCR and Western blot confirmed the acquisition of an EMT phenotype. Thus, significant upregulation of *ZEB1*, *VIM*, and *SNAI2* was found in both resistant sublines (Figure 2E). *CDH2* was also significantly upregulated in NOZ GemR cells (*p* = 0.0211) but downregulated in TGBC1 GemR cells (*p* = 0.0083) (Figure 2E). On the other hand, transcript levels of *SNAI1* increased in TGBC1 GemR cells but decreased in NOZ GemR cells (*p* = 0.0105) (Figure 2E). P-values and fold-change comparisons between microarray data and qRT-PCR are detailed in Tables S6 and S7. Changes in protein levels showed agreement with gene expression (Figure 2F and Figure S3). N-cadherin was undetectable by Western blot, probably because transcript levels were close to the Ct cut-off value.

Alteration of drug metabolism was also identified as a potential chemoresistance mechanism. In particular, we found a significant upregulation of *CDA* in both resistant cell sublines (*p* = 0.0033 NOZ GemR and *p* = 0.0091 TGBC1 GemR), although the fold change was less than 3 in TGBC1 GemR cells (2.32, Table S7). *CDA* encodes cytidine deaminase, an enzyme capable of inactivating cytidine nucleoside analogs and thereby limiting the antineoplastic efficacy of gemcitabine [17]. *CDA* was also identified as a dysregulated xenobiotic-metabolism gene in NOZ GemR according to the pathway-driven and leading-edge analyses (Figure 2A,D). Further validation by qRT-PCR and Western blot showed a significant upregulation and overexpression of CDA in both gemcitabine-resistant sublines compared to parental cells, being higher in NOZ GemR cells (*p* = 0.0007) than in TGBC1 GemR cells (*p* = 0.0039) (Figure 2E,F).

We also evaluated the gene expression of drug transporters involved in the cellular uptake and efflux of gemcitabine since they have been linked to chemoresistance and low clinical responses. Both gemcitabine-resistant cells exhibited a significant downregulation of *SLC29A1* and *SLC29A2*, genes encoding ENT1 and ENT2, respectively, the major transporters involved in the cellular uptake of gemcitabine (Figure S4). None of the efflux ABC transporters were upregulated in NOZ GemR cells, whereas *ABCC2* was significantly upregulated in TGBC1 GemR cells (*p* = 0.0308) (Figure S4), which could explain the cross-resistance to 5-FU and cisplatin observed in TGBC1-resistant cells.

Taken together, our analyses show that gemcitabine-resistant GBC cells carried out transcriptional reprograming consistent with a chemoresistance phenomenon, which seems to be associated with the acquisition of mesenchymal features and the dysregulation of processes associated with the metabolism of xenobiotics.

### 2.3. Dysregulation of Gemcitabine Metabolism-Related Genes Is an Early Adaptation of GBC Cells to Drug Toxicity

CDA was the main gemcitabine metabolism-related enzyme found to be upregulated in both stable drug-resistant GBC cell models. Thus, we wanted to clarify whether *CDA* upregulation is an early cell adaptation in response to gemcitabine-induced stress. We extended the analysis to ENT and ABC transporters and evaluated four GBC cell lines exhibiting varying sensitivity to gemcitabine, from the most sensitive to the most resistant (NOZ, G-415, TGBC1, and TGBC2) (Table S8, Figure S5).

**Figure 3:**
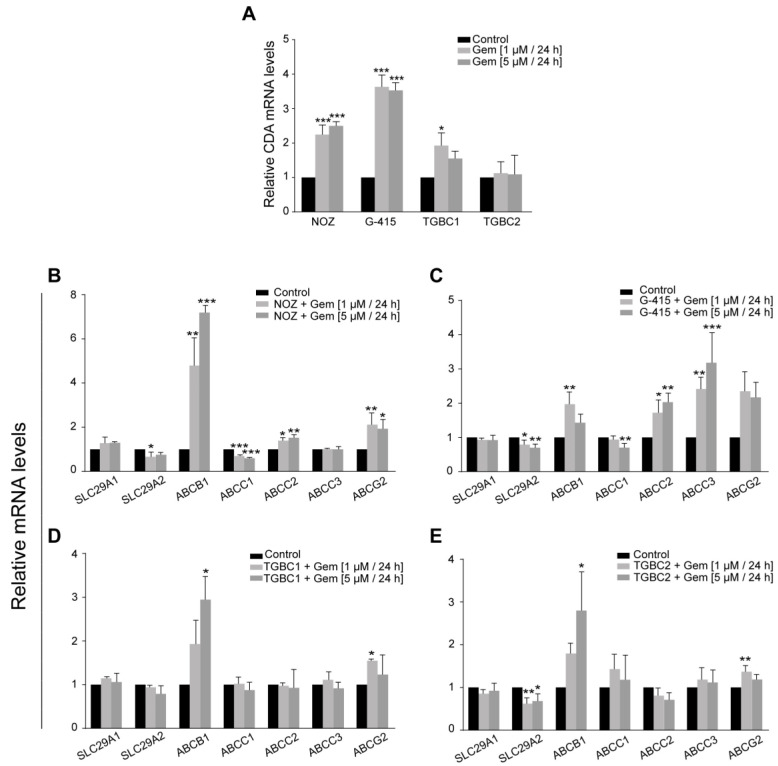
Differential gene expression induced by short-term exposure to gemcitabine. (**A**) Relative expression of *CDA*; and (**B**–**E**) the relative expression of genes encoding ENT1/2 and ABC transporters. Relative gene expression was normalized to *TFCP2* and *QARS* (mean ± SD). All data are representative of three independent experiments (* *p* < 0.05, ** *p* < 0.01, and *** *p* < 0.001 by one-way ANOVA or Kruskal–Wallis test).

The modulation of gene expression was evaluated by comparing Ct values (2^−ΔΔCt^) between treated and nontreated cells after exposing them to high doses of gemcitabine (1 and 5 µM) for 24 h. A significant upregulation of *CDA* was observed in three cell lines, but the fold change was higher in the most sensitive cell lines, NOZ and G-415 (Figure 3A).

All GBC cell lines exhibited the significant upregulation of at least two drug efflux transporters after gemcitabine treatment, with the upregulation of *ABCB1* being a common event in all of them (Figure 3B–E). As expected, TGBC1 and TGBC2 showed minor changes as they intrinsically already express high levels of ABC transporters compared to NOZ and G-415 (Figure S5). Regarding the ENT transporters, the levels of *SLC29A2* transcripts decreased significantly in NOZ (*p* = 0.0231 at 1 µM), G-415 (*p* = 0.0374 at 1 µM and *p* = 0.0093 at 5 µM), and TGBC2 (*p* = 0.0050 at 1 µM and *p* = 0.0195 at 5 µM) after gemcitabine treatment, whereas no significant changes were observed in *SLC29A1* expression (Figure 3B–E).

These results suggest that GBC cells could avoid gemcitabine cytotoxicity by activating mechanisms associated with drug transport and metabolism. Among those events, the upregulation of *CDA* seems to be an early adaptation to acute drug stress (24 h), which is retained during the acquisition of stable gemcitabine resistance.

### 2.4. Quantitative Analysis of Tyrosine Phosphoproteome in Gemcitabine-Resistant NOZ Cells

**Figure 4:**
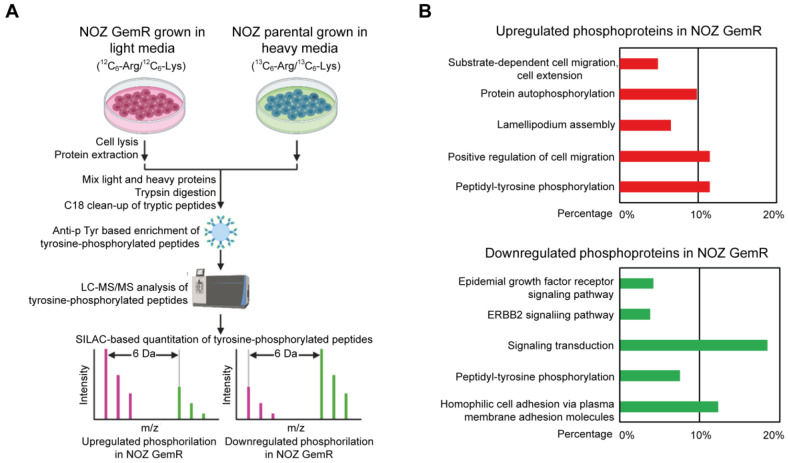
Dysregulated tyrosine phosphorylation in NOZ GemR cells quantified by SILAC. (**A**) SILAC quantitation experimental design. “Light” NOZ GemR cells (purple) and “heavy” NOZ parental cells (green) were lysed, mixed, and subjected to in-solution trypsin digestion. The tryptic peptides were enriched by an anti-phosphotyrosine antibody for tyrosine-phosphorylated peptides. The enriched phosphopeptides were analyzed on an Orbitrap Fusion Lumos Tribrid mass spectrometer. Upregulated and downregulated phosphorylation in NOZ GemR cells is revealed by the ratio of paired SILAC peaks. (**B**) GO biological process terms specific to upregulated (upper) and downregulated (bottom) phosphoproteins in NOZ GemR cells. The bars represent the percentage of proteins associated with the terms.

To identify signaling molecules associated with acquired gemcitabine resistance in GBC, we combined SILAC-based quantitative proteomics with an anti-phosphotyrosine-based enrichment method to quantify the difference in tyrosine phosphorylation between parental NOZ and NOZ GemR cells (Figure 4A). From duplicate experiments, 1659 tyrosine phosphorylation sites were quantified on 983 proteins. Specifically, we identified 61 proteins with 83 tyrosine hyperphosphorylation sites in NOZ GemR cells and 211 proteins with 320 tyrosine hyperphosphorylation sites in parental NOZ cells. The phosphosites are summarized in Tables S9–S11.

Proteins with increased tyrosine phosphorylation in NOZ GemR cells were significantly enriched in multiple biological processes including peptidyl-tyrosine phosphorylation, the positive regulation of cell migration, lamellipodium assembly, protein autophosphorylation, and substrate-dependent cell migration/cell extension (Figure 4B, Table S12). In the case of parental NOZ cells, proteins with increased tyrosine phosphorylation were significantly enriched in homophilic cell adhesion via plasma membrane adhesion molecules, peptidyl-tyrosine phosphorylation, signal transduction, the ERBB2 signaling pathway, and the EGFR signaling pathway (Figure 4B, Table S13).

To predict the kinase activity in NOZ parental and NOZ GemR cells, we first listed the kinases with increased phosphosites and determined the kinase-regulatory phosphosites using the information registered in Uniprot and PhosphoSitePlus (Table S14). The activation of growth factor receptors (AXL, EGFR, and MET) in parental NOZ cells was consistent with our observation that parental cells grow faster than corresponding gemcitabine-resistant cells. On the other hand, three tyrosine kinases were potentially activated in NOZ GemR cells: ABL1, LYN, and PDGFR. ABL1 was hyperphosphorylated at three activation phosphosites (Y226, Y393, and Y185) and its substrates also showed increased phosphorylation (ABL interactor 1 (ABL1) at Y213) and vinculin (VCL) at Y822). Among the LYN substrates, cortactin (CTTN) was found hyperphosphorylated at Y421 and Y446 in NOZ GemR cells. We also found the increased phosphorylation of multiple PDGFR phosphotyrosine residues (Y742, Y768, Y762, Y849, and Y1017) that act as docking sites and are required for PDGFR interaction with the signaling molecules PIK3R1, CRK, and PLCG1.

### 2.5. Dasatinib Selectively Killed NOZ GemR Cells and Reduced Their Migration Ability

**Figure 5:**
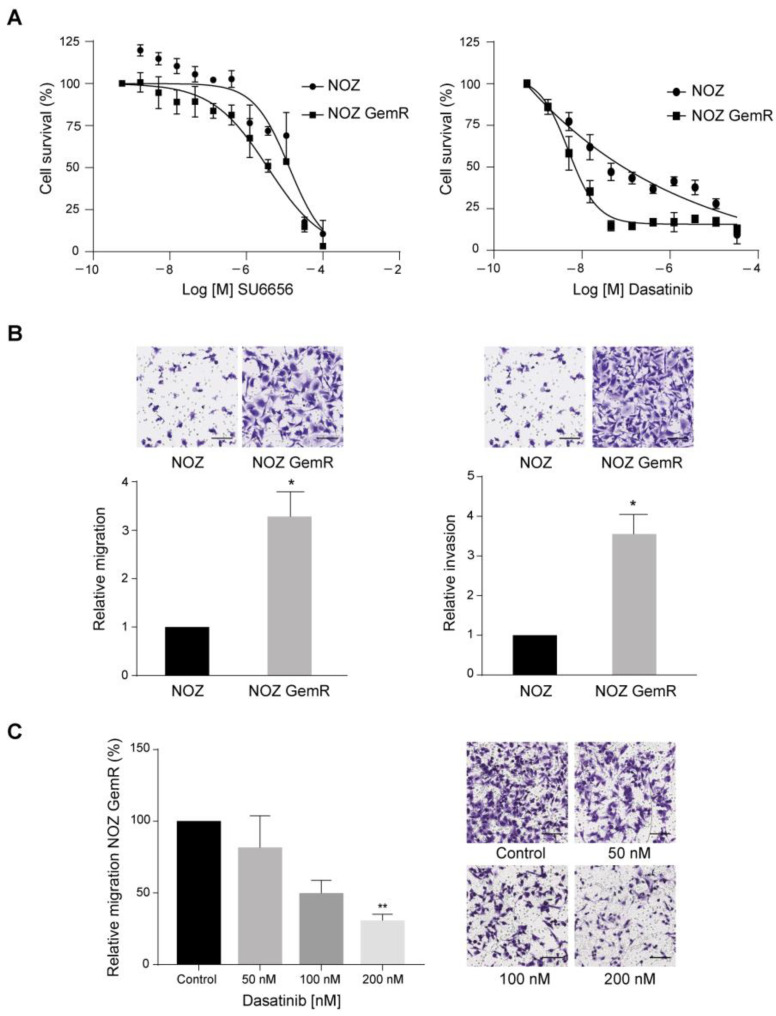
The in vitro effect of dasatinib on cell viability and the migration of NOZ GemR cells. (**A**) Dose-response curves of NOZ GemR and NOZ parental cells after 72 h of incubation with dasatinib and SU6656 (mean ± SD); (**B**) differential migratory and invasive capacity of NOZ GemR cells compared to NOZ parental cells (mean ± SD); and (**C**) relative cell migration in NOZ GemR cells after treatment with dasatinib (mean ± SD). Representative images: 10× magnification, scale bar: 250 µm. All data are representative of three independent experiments with three technical replicates. (* *p* < 0.05; ** *p* < 0.01 using the Kruskal–Wallis test).

We reasoned that one or more of the active kinase candidates could be used as potential therapeutic targets for overcoming chemoresistance to gemcitabine in gallbladder cancer. We chose two inhibitors to test the dependency of NOZ GemR cells on the potentially activated pathways: SU6656, a small molecule that selectively inhibits members of the Src kinase family [18], and dasatinib, which is a potent tyrosine kinase inhibitor that targets multiple kinases including ABL, Src family kinases, and PDGFRA [19]. Those agents interact with the active site of their target kinases when they are actively phosphorylated, affecting the downstream signaling. Therefore, we sought to examine the antiproliferative effects of these two inhibitors in NOZ parental and NOZ GemR cells. As shown in Figure 5A, NOZ GemR cells were more sensitive to each inhibitor compared to parental cells; however, the potency of dasatinib was higher than SU6656, with an IC_50_ of ~5 nM after 72 h of treatment.

The acquired gemcitabine resistance was linked to a more migratory and invasive phenotype in NOZ GemR cells (Figure 5B). After 24 h, we found that NOZ GemR cells exhibited a significant 3.2-fold (*p* = 0.029) and 3.5-fold (*p* = 0.029) increase in their ability to migrate and invade, respectively. We then explored the effect of dasatinib on the in vitro migratory and invasive capacity of NOZ GemR cells using inhibitor concentrations that did not significantly affect cell viability (Figure S6). Transwell inserts were prepared, as described in the Methods section, but cells were suspended in a serum-free medium containing the vehicle or the inhibitor at the indicated concentrations. As shown in Figure 5C, the migration of NOZ GemR cells was reduced by 18%, 49%, and significantly reduced by 69% (*p* = 0.004) at 50, 100, and 200 nM, respectively, compared to the vehicle control. No differences were observed in cell invasion (Figure S7). Taken together, our results indicated that dasatinib can effectively hinder the migration of NOZ GemR cells.

## 3. Discussion

Gallbladder cancer is an aggressive disease with few therapeutic options, low treatment response rates, and high recurrence risk [1]. The chemotherapeutic failure in many cancers is largely attributed to drug resistance, a complex and multifactorial process that allows tumor cells to survive therapy due to drug-induced selective pressure and adaptive tumor evolution [20]. Currently, the underlying mechanisms associated with the development of chemoresistance in GBC have been scarcely explored.

Here, we investigated the potential molecular pathways involved in acquired resistance to gemcitabine in GBC by developing two gemcitabine-resistant cell lines (NOZ GemR and TGBC1 GemR). Our in vitro models of acquired gemcitabine resistance shared morphological and molecular features, even though the parental cells exhibited different sensitivity to the drug. EMT was a particularly enriched biological process in both GemR cells. As is known, during EMT, tumor cells shift between epithelial and mesenchymal states, acquiring stem cell-like properties that are ultimately responsible for increased drug resistance, invasiveness, and metastatic ability [21]. Here, the dysregulation of several EMT-related genes as well as the spindle-shaped morphology and increased cell size observed in both gemcitabine-resistant GBC sublines supported the acquisition of mesenchymal traits similar to that observed in pancreatic tumor cells with acquired resistance to gemcitabine [22,23]. The transcription factors that mediate gene expression reprogramming during EMT (EMT-TFs) include the zinc-finger Snail homologs (Snai1/Snail, Snai2/Slug, and Snai3), zinc-finger E-box-binding (ZEB1 and ZEB2/SIP1) and several basic helix-loop-helix factors such as Twist and TCF3/E47/E12 [24]. In this study, we report increased levels of EMT-TFs in GemR cells compared to respective parental cells, such as Slug and ZEB1. Slug induces vimentin expression [25] and acts as a transcriptional repressor of *CDH1* [26]. Accordingly, vimentin expression was higher in gemcitabine-resistant sublines, and E-cadherin expression was reduced in TGBC1 GemR cells. The dysregulation of EMT-TFs in GBC has not been evaluated in the context of chemoresistance; however, the overexpression of ZEB1 in GBC tissues has been associated with aggressive traits, such as peritumoral tissue invasion [27].

The EMT-induced chemoresistance can be mediated by the upregulation of ATP-binding cassette (ABC) transporters. For instance, MDR1 expression has been regulated by Twist in a model doxorubicin-resistant liver cancer cell line (HepG2) [28] and by Snail in colorectal cancer cells [29]. The downregulation of ENT1, the main transporter regulating the cellular uptake of gemcitabine, has also been pointed to as an EMT-mediated chemoresistance mechanism, as recently demonstrated in pancreatic tumor cells, where the E- to N-cadherin switching initiated by EMT negatively influenced the expression of ENT1, cell surface location, and transport function, leading to gemcitabine resistance [30]. In our study, only TGBC1 GemR cells exhibited the significant upregulation of ATP-transporters coding genes, specifically *ABCB1* (MDR1) and *ABCC2* (MRP2), together with the overexpression of Snail, Slug, and ZEB1. By contrast, in NOZ GemR cells, none of the five ABC transporters evaluated showed significant changes in gene expression, although the overexpression of Slug was also observed. Regarding ENT1 expression, only NOZ GemR cells exhibited downregulation of the ENT1 coding gene *SLC29A1*. Taken together, our results suggest a major role of EMT in gemcitabine resistance in GBC; however, further analyses are needed to clarify the functional role and regulatory networks of EMT-related transcription factors during the acquisition of drug resistance in GBC.

The efficacy of gemcitabine is limited due to CDA-mediated detoxification [31]. In pancreatic cancer cell lines, CDA directly metabolizes the captured gemcitabine (dFdC) to an inactive form (dFdU), decreasing the cytoplasmic concentration of phosphorylated gemcitabine [32]. High levels of CDA have been related to the development of gemcitabine resistance in breast [33] and pancreatic tumor cells [34,35,36]. Here, we demonstrated a significant increase in CDA expression in both gemcitabine-resistant sublines, as well as increased transcript levels in GBC cell lines exposed to short-term gemcitabine treatment. Higher changes in *CDA* expression were found in gemcitabine-sensitive GBC cells (NOZ and G-415), probably because the most resistant cells (TGBC1 and TGBC2) were able to avoid cytotoxicity through enhanced drug efflux since the ABC transporters were upregulated in those cells. Although our results suggest that CDA overexpression could be a protective mechanism of GBC cells against gemcitabine, thereby enhancing drug detoxification processes, additional experiments are needed to determine the role of CDA in the early adaptation of GBC cells to gemcitabine-induced stress, including the assessment of protein expression and functional analyses. To the best of our knowledge, these findings have not been reported in GBC; however, CDA has been proposed as a prognostic factor [37], therapeutic target [38], or predictive biomarker for cytotoxicity [39], and therapy response [40] against gemcitabine-based treatments in other tumors.

Finally, we employed quantitative phosphoproteomics analysis to identify aberrantly activated signaling events associated with acquired gemcitabine resistance in NOZ cells, which may contribute to clarifying novel druggable targets. Our analyses revealed the potential activation of three kinases in NOZ GemR cells: ABL1, PDGFRA, and LYN. These tyrosine kinases play a role in multiple key cellular processes, and their dysregulation has been associated with the aggressive behavior of tumor cells. For instance, ABL kinases are involved in the regulation of actin remodeling through the tyrosine phosphorylation of proteins controlling cytoskeleton dynamics [41]. The activation of ABL kinases has been shown to regulate the expression of EMT transcription factors and promote tumor invasion in melanoma [42]. LYN, a member of the Src kinase family of nonreceptor tyrosine kinases (SFKs), transmits signals from cell surface receptors and controls the regulation of several biological processes [43]. LYN kinase activity has been reported as significantly elevated in other solid tumors, such as pancreatic [44] and breast cancer [45,46]. PDGFRA, on the other hand, is a cell surface tyrosine kinase receptor that plays an essential role in the regulation of embryonic development, cell proliferation, survival, and chemotaxis [47]. The pharmacological inhibition of those kinases using dasatinib, a multikinase inhibitor, resulted in the decreased viability and cell migration of NOZ GemR cells. The specificity of dasatinib on gemcitabine-resistant cells compared to parental cells suggest that one or more of the targeted pathways are activated in GemR cells and could represent a therapeutic vulnerability in gallbladder cancer having acquired resistance to chemotherapy. Previous reports have shown that dasatinib reduces cell motility and increases tumor chemosensitivity by proapoptotic mechanisms and the reduction in cancer stemness, respectively [48,49]. In GBC, dasatinib reduced tumor growth in vivo and enhanced the therapeutic response to anti-EGFR treatment [50]. Therefore, dasatinib could represent an effective pharmacological strategy to reverse gemcitabine resistance in GBC, although validation in preclinical models is necessary to confirm the activation of those kinases (ABL1, PDGFRA, and LYN) in gemcitabine-resistant tumors and the effectiveness of dasatinib to potentially overcome resistance to chemotherapy in GBC.

In summary, the transcriptomic and phosphoproteomic characterization of gemcitabine-resistant GBC cells identified multiple potential biomarkers for predicting and/or monitoring chemoresistance and uncovered potential molecular pathways that could be targeted to overcome the resistant phenotype. For instance, the increased expression of *CDA*, *FN1*, *LAMC2*, and EMT-related markers, as well as potentially activated tyrosine kinases. Thus, these two drug-resistant GBC sublines may be useful in exploring and validating potential therapeutic targets for chemotherapy-refractory GBC.

## 4. Materials and Methods

### 4.1. Cell Culture

The human GBC cell line NOZ was obtained from the Health Science Research Resources Bank (Osaka, Japan; No. JCRB1033). The other three GBC cell lines, G-415, TGBC-1TKB (TGBC1), and TGBC-2TKB (TGBC2), were purchased from RIKEN BioResource Center (Ibaraki, Japan; No. RCB2640, RCB1129, and RCB1130). NOZ, TGBC1, and TGBC2 were grown in Dulbecco’s Modified Eagle Medium (DMEM high glucose; Corning, NY, USA) supplemented with 5% fetal bovine serum (FBS, Thermo Fisher Scientific Inc., Waltham, MA, USA) and 10 units/mL penicillin and 10 mg/mL streptomycin (1% P/S, Thermo Fisher Scientific). The G-415 cells were grown in RPMI 1640 medium (Thermo Fisher Scientific) supplemented with 10% FBS and 1% P/S. Cells were maintained at 37 °C in a humidified atmosphere containing 5% CO_2_. All cell lines were routinely tested for mycoplasma by PCR and authenticated by short tandem repeat DNA profiling.

### 4.2. Establishment of Gemcitabine-Resistant Sublines

Gemcitabine-resistant sublines (NOZ GemR and TGBC1 GemR) were established by exposing parental cell lines to stepwise increased gemcitabine (Sigma-Aldrich, St. Louis, MO, USA) concentrations. In brief, cells at subconfluent densities were exposed to low doses of gemcitabine starting at absolute ~IC_80_ (2 nM for NOZ and 5 nM for TGBC1, as determined from the dose-response curves). At every two subcultures at 70% confluence, the concentration of gemcitabine was gradually increased until it reached 500 nM. The establishment of a stable gemcitabine-resistant subline was determined by comparing the IC_50_ values of parental and resistant cells after growing them in a gemcitabine-free medium for at least 1 month. The resistance index (RI) was determined as the ratio of the IC_50_ values between the resistant and parental cells. The stability of drug resistance was examined, when necessary, at monthly intervals.

### 4.3. Measurement of Cell Viability

Cells were seeded into 96-well plates at a density of 3.5 × 10^3^ cells/well in 100 μL of cell culture medium. After an overnight attachment period, the cells were treated for 72 h with a serial dilution of the drug, and cell viability was assessed by incubating the cells for 2 h at 37 °C with MTS–PMS colorimetric solution (Promega Corp., Madison, WI, USA). The 11-point 1:3 serial dilutions started from 300 µM for gemcitabine, 500 µM for cisplatin (Calbiochem, Merck Group, Darmstadt, Germany), 2000 µM for 5-fluorouracil (5-FU, Laboratorios Kampar, Santiago, Chile), and 100 µM for SU6656 (Calbiochem) and dasatinib (Selleckchem, Houston, TX, USA). Absorbance was read at a wavelength of 490 nm using the Epoch™ microplate spectrophotometer (BioTek Instruments Inc., Winooski, VT, USA). The half-maximal inhibitory concentration (IC_50_) was calculated from the dose-response curves. Three independent experiments were performed with three technical replicates for each.

### 4.4. Cell Growth Characteristics

Doubling time (DT) was calculated by seeding 10,000 cells per well in 12-well plates and counting them using Trypan blue dye exclusion with a Neubauer camera (Marienfeld-Superior, Lauda-Königshofen, Germany) every 24 h for 4 days. The population doubling time was determined from the growth curves using the “cell calculator++” online tool (https://doubling-time.com/compute_more.php (accessed on 16 April 2018). Three independent experiments were performed with three technical replicates for each.

### 4.5. Transwell Migration and Invasion Assays

A total of 1 × 10^4^ and 4 × 10^4^ cells (for migration and invasion, respectively) were seeded into the upper chamber of 8 µm-pore transwell inserts (Corning, Corning, NY, USA) in serum-free DMEM and allowed to migrate/invade for 24 h toward the lower chamber containing 600 µL of DMEM with 10% FBS. In the case of the invasion assays, the transwell inserts were precoated with 20 µg of Matrigel (Corning, NY, USA). At the end of the incubation period, the membranes were removed from the inserts using a scalpel blade, mounted onto a glass slide, and scanned using the Aperio Digital Pathology Slide Scanner AT2 (Leica Biosystems, Wetzlar, Germany). The number of cells that migrated/invaded was counted in at least five randomly selected fields at 10× magnification (ImageJ v1.5, National Institutes of Health, Bethesda, MD, USA). Three independent experiments were performed in duplicate for each assay.

### 4.6. Total RNA Extraction and Microarray Hybridization

Total RNA was extracted using the AllPrep DNA/RNA/miRNA Universal Kit (Qiagen, Valencia, CA, USA) and quantified by spectrophotometry on the Epoch™ microplate spectrophotometer. RNA quality was assessed using the 2200 TapeStation System (Agilent Technologies, Santa Clara, CA, USA). A total of 500 ng of RNA was used to synthesize double-stranded cDNA and the corresponding in vitro transcription cRNA (Ambion WT Expression Kit, Thermo Fisher Scientific). Subsequently, cRNA was purified, quantified, and subjected to single-strand (ss) cDNA synthesis. The purified ss cDNA was then fragmented to <150 bp and the end-terminus was labeled using GeneChip^TM^ WT Terminal Labeling Kit (Thermo Fisher Scientific) following the manufacturer’s guidelines. The labeled samples were then hybridized at 45 °C for 16 h to the Affymetrix human transcriptome array 2.0 according to the guidelines of the Affymetrix GeneChip WT Terminal Labeling and Hybridization User Manual (Thermo Fisher Scientific). Three independent experiments were performed.

### 4.7. Microarray Data Analysis

The CEL files generated by the Affymetrix GeneChip^®^ Command Console (AGCC) were imported into the Affymetrix Transcriptome Expression Console (TAC) software (version 4.0.2.15) for data normalization using the default robust multichip analysis (SST-RMA) algorithm. Normalized data were then analyzed using the Gene Level Differential Expression Analysis function to identify differentially expressed protein-coding genes with an ANOVA *p*-value cut-off of <0.01 and a fold change of ≥3. ClueGO was used for gene ontology (GO) analysis [51], considering a kappa score level threshold of 0.4 for the term–term interrelation and including only clusters with a *p*-value of <0.001 and a Bonferroni correction. Gene set enrichment analysis (GSEA) in preranked mode [16] was performed using differentially expressed genes (*p*-value cut-off of <0.01) that were tested against a collection of 50 hallmark gene sets available in MSigDB, v7.5.1 (www.gsea-msigdb.org; accessed on 3 August 2022). A leading-edge analysis was then performed in the GSEA software (version 4.2.2) to overlay the gene subsets that contribute the most to the enrichment of the top five dysregulated biological pathways and thus identify the upregulated genes shared among those biological processes.

### 4.8. Quantitative Real-Time PCR Analysis (qRT-PCR)

Complementary DNA (cDNA) synthesis was carried out from 1 µg of RNA using the AffinityScript QPCR cDNA Synthesis Kit (Agilent). Then, quantitative PCR was performed using the 2× Brilliant II SYBR^®^ Green qPCR master mix (Agilent) and the Cobas Z480 Analyzer (Roche, Indianapolis, IN, USA). Three biological replicates were performed for each experimental condition and the relative expression levels were quantified using the 2^−ΔΔCt^ method [52] and using *TFCP2* and *QARS* as normalizers. Primer sequences are shown in Table S15.

### 4.9. Western Blot Analysis

Protein expression was analyzed by Western blot assays, as previously described [53]. At least three biological assays were performed. A summarized protocol is shown in the Supplementary Materials: Supplementary Methods/Western blot analysis. The following rabbit monoclonal antibodies were used in this study: anti-Vimentin (D21H3, #5741), anti-E-cadherin (24E10, #3195), anti-Snail (C15D3, #3879), anti-Slug (C19G7, #9585), anti-N-cadherin (D4R1H, #13116), and anti-β-Actin (13E5, #4970) from Cell Signaling Technologies Inc. (Danvers, MA, USA); anti-CDA (EPR20525, #ab222515) from Abcam (Cambridge, UK); and anti-Zeb1 (#HPA027524) from Sigma-Aldrich. HRP-conjugated goat anti-rabbit (#7074) secondary antibody was obtained from Cell Signaling Technologies Inc.

### 4.10. SILAC Labeling, Peptide Preparation, and Phosphopeptide Enrichment

NOZ cells were grown in a ^13^C_6_-lysine/^13^C_6_-arginine-containing (heavy) medium, while NOZ GemR cells were grown in a normal (light) medium. The experiment was carried out in a biological duplicate. DMEM with and without lysine and arginine, fetal bovine serum (FBS), L-glutamine, and antibiotics were purchased from Thermo Fisher Scientific. SILAC amino acids, ^13^C_6_-lysine, and ^13^C_6_-arginine were acquired from Cambridge Isotope Laboratories (Andover, MA, USA). Peptides were prepared using an in-solution tryptic digestion protocol with modifications [54,55]. The eluted peptides were lyophilized and subjected to phosphopeptide enrichment by immunoaffinity purification (IAP), as previously described [54,55]. Peptides were eluted twice from beads by incubating the beads with 0.1% TFA at room temperature. Protocol details are shown in the Supplementary Materials: Supplementary Methods/SILAC protocol.

### 4.11. Liquid Chromatography–Tandem Mass Spectrometry (LC–MS/MS)

The LC–MS/MS analysis of IAP-enriched phosphopeptides was carried out using a reversed-phase liquid chromatography system interfaced with an Orbitrap-equipped Fusion Lumos Tribrid mass spectrometer. The peptides were loaded onto an analytical column (10 cm × 75 cm, Magic C18 AQ 5 µm, 120 Å) with 0.1% formic acid and eluted using an acetonitrile gradient (0–60%) containing 0.1% formic acid. The settings were: (a) precursor scans (FTMS) from 350–1550 *m*/*z* at a 120,000 resolution; and (b) MS2 scan (FTMS) of HCD fragmentation of the most intense ions (cycle time: 30 s; isolation mode: quadrupole; isolation width: 1.60 *m*/*z*; Isolation *m*/*z* offset: 0.3; stepped collision energy (%): 5; collision energy (%): 32; activation Q = 0.25; FT first mass value: 110.00 (fixed); and data type: centroid at a 30,000 resolution.

### 4.12. Mass Spectrometric Data Analysis

The tandem mass spectra were searched using the Andromeda algorithm against a human UniProt database (2020-06 release) through the MaxQuant platform (version 1.6.17.0). The search parameters included: SILAC 2-state, Arg6/Lys6; a maximum of three SILAC labels per peptide; a maximum of two missed cleavages; fixed modification: carbamidomethylation of cysteine; variable modification: protein N-term acetylation, oxidation of methionine, deamination of asparagine and glutamine, and the phosphorylation of serine, threonine, and tyrosine. See the Supplementary Materials: Supplementary Methods/Mass spectrometric data analysis for details on parameter values. The quantification of each identified phosphosite was calculated by MaxQuant. The probability of phosphorylation for each Ser/Thr/Tyr site on each peptide was calculated using Andromeda (MaxQuant). For each identified tyrosine phosphorylation site, if the ratio of NOZ GemR over NOZ was more than 2 or there was a lack of heavy peak in both replicates, the site was considered upregulated in NOZ GemR cells; on the other hand, if the ratio of NOZ GemR over NOZ was less than 0.5 or there was lack of light peak in both replicates, the site was considered downregulated in NOZ GemR cells. The GO analysis of up- and downregulated proteins in the biological process was carried out in DAVID Bioinformatics Resource [56,57].

### 4.13. Statistical Analysis

Data were statistically analyzed using GraphPad Prism 8 software for Windows (GraphPad, San Diego, CA, USA). Between-group comparisons were analyzed using Wilcoxon–Mann–Whitney U or Kruskal–Wallis tests. Expression levels from qPCR data were first analyzed using the Shapiro–Wilk test to determine normality. Then, a two-tailed Student’s *t*-test with Welch’s correction or Wilcoxon–Mann–Whitney U test was carried out to compare two groups, while a one-way ANOVA or Kruskal–Wallis test was applied to compare mRNA levels among more than two independent groups. For all tests, differences were taken to be statistically significant at a *p*-value < 0.05 (* *p* < 0.05, ** *p* < 0.01, *** *p* < 0.001, and **** *p* < 0.0001).

## Data Availability

Transcriptomics data have been submitted to the Gene Expression Omnibus database under the accession numbers GSE208659 (NOZ and NOZ GemR) and GSE208660 (TGBC1 and TGBC1 GemR); proteomics data are available at ProteomeXchange via identifier PXD036348.

## Supplementary Materials

The following supporting information can be downloaded at: https://www.mdpi.com/article/10.3390/ijms24087238/s1.
