# Supplementary material for: A Novel Gemcitabine-Resistant Gallbladder Cancer Model Provides Insights into Molecular Changes Occurring during Acquired Resistance
Source: Int J Mol Sci. 2023 Apr 14;24(8):7238. doi: 10.3390/ijms24087238 (PMC10139168; doi:10.3390/ijms24087238)
Supplement: Supplementary file 1 [file ijms-24-07238-s001.zip › Supplementary material.pdf]

International Journal of
Molecular Sciences

MDPI

Supplementary Materials

A Novel Gemcitabine-Resistant Gallbladder Cancer Model Provides Insights into Molecular Changes Occurring during Acquired Resistance

Table S1. Half maximal inhibitory concentration (IC_{50}) values to cytotoxic drugs and resistance index (RI) for parental and gemcitabine-resistant cells.

	Chemotherapeutic (μM)		
**Cell line**	**Gemcitabine**	**Cisplatin**	**5-Fluorouracil**
NOZ	0.006	2.88	1.45
NOZ GemR	0.203	3.05	1.78
**RI**	**0.033**	**1.06**	**1.23**
TGBC1	0.040	2.21	8.93
TGBC1 GemR	0.891	6.43	211.35
**RI**	**0.022**	**2.90**	**23.6**

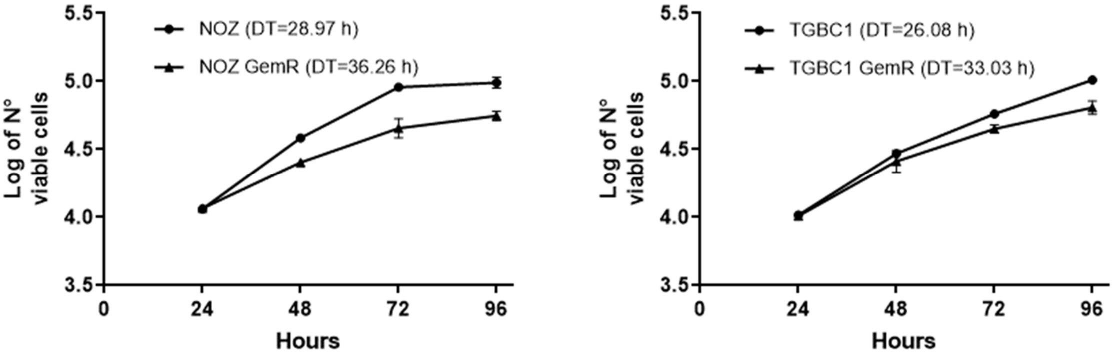

Figure S1. Proliferation curves of parental and gemcitabine-resistant cells. Viable cells were counted by Trypan Blue dye exclusion and doubling time (DT) was estimated using the Doubling Time Software. Values are the means ± SD of three independent experiments, each with three technical replicates.

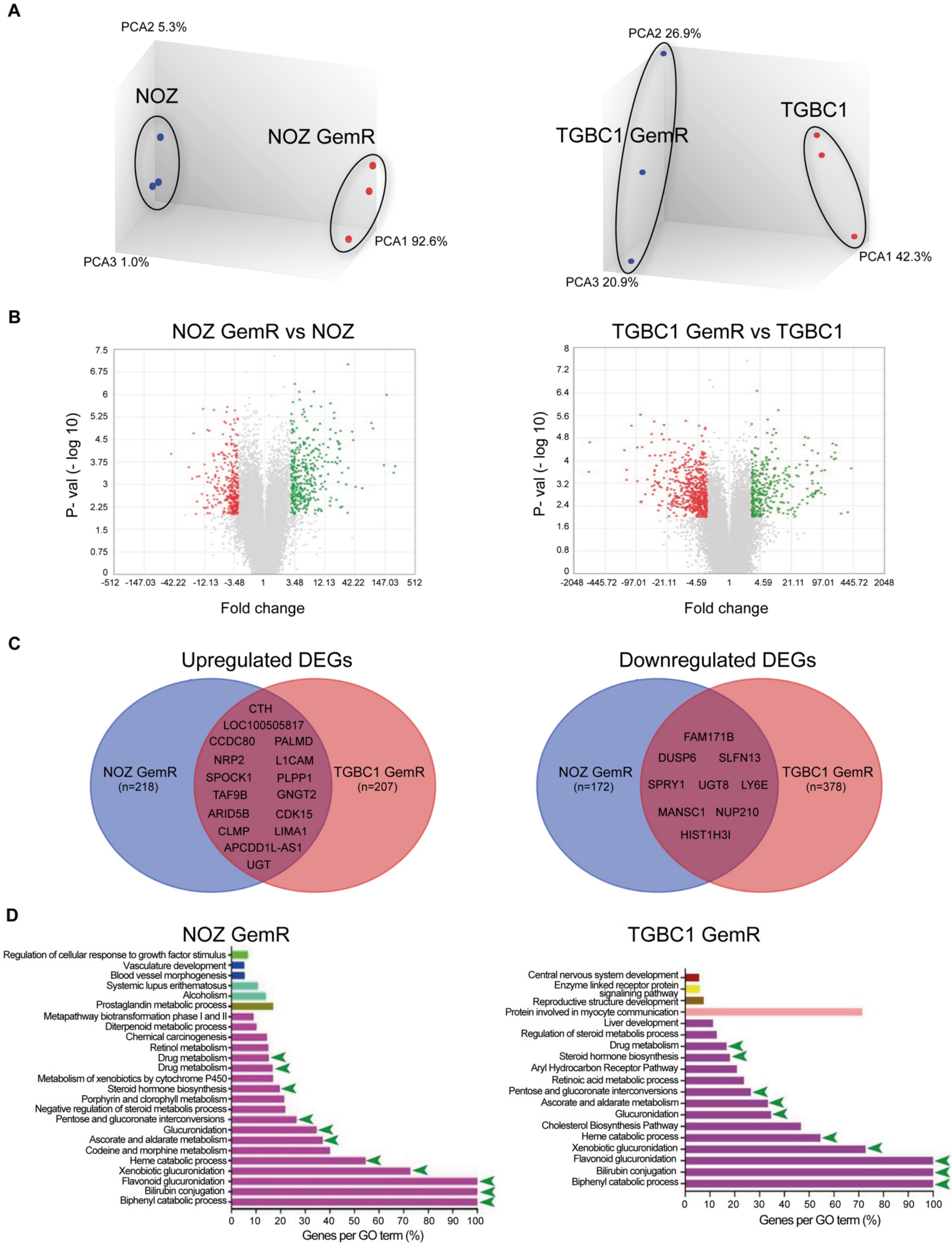
A
PCA2 5.3%
NOZ
NOZ GemR
PCA3 1.0%
PCA1 92.6%
PCA2 26.9%
TGBC1
TGBC1 GemR
PCA3 20.9%
PCA1 42.3%
B
NOZ GemR vs NOZ
P- val (- log 10)
Fold change
-512 -147.03 -42.22 -12.13 -3.48 1 3.48 12.13 42.22 147.03 512
TGBC1 GemR vs TGBC1
P- val (- log 10)
Fold change
-2048 -445.72 -97.01 -21.11 -4.59 1 4.59 21.11 97.01 445.72 2048
C
Upregulated DEGs
NOZ GemR (n=218)
TGBC1 GemR (n=207)
CTH
LOC100505817
CCDC80 PALMD
NRP2 L1CAM
SPOCK1 PLPP1
TAF9B GNGT2
ARID5B CDK15
CLMP LIMA1
APCDD1L-AS1
UGT
Downregulated DEGs
NOZ GemR (n=172)
TGBC1 GemR (n=378)
FAM171B
DUSP6 SLFN13
SPRY1 UGT8 LY6E
MANSC1 NUP210
HIST1H3I
D
NOZ GemR
Regulation of cellular response to growth factor stimulus
Vasculature development
Blood vessel morphogenesis
Systemic lupus erithematosus
Alcoholism
Prostaglandin metabolic process
Metapathway biotransformation phase I and II
Diterpenoid metabolic process
Chemical carcinogenesis
Retinol metabolism
Drug metabolism
Drug metabolism
Metabolism of xenobiotics by cytochrome P450
Steroid hormone biosynthesis
Porphyrin and clorophyll metabolism
Negative regulation of steroid metabolis process
Pentose and glucoronate interconversions
Glucuronidation
Ascorate and aldarate metabolism
Codeine and morphine metabolism
Heme catabolic process
Xenobiotic glucuronidation
Flavonoid glucuronidation
Bilirubin conjugation
Biphenyl catabolic process
Genes per GO term (%)
TGBC1 GemR
Central nervous system development
Enzyme linked receptor protein signalining pathway
Reproductive structure development
Protein involved in myocyte communication
Liver development
Regulation of steroid metabolis process
Drug metabolism
Steroid hormone biosynthesis
Aryl Hydrocarbon Receptor Pathway
Retinoic acid metabolic process
Pentose and glucoronate interconversions
Ascorate and aldarate metabolism
Glucuronidation
Cholesterol Biosynthesis Pathway
Heme catabolic process
Xenobiotic glucuronidation
Flavonoid glucuronidation
Bilirubin conjugation
Biphenyl catabolic process
Genes per GO term (%)

Figure S2. Transcriptomic analyses of gemcitabine-resistant cells compared to parental cells. A) Principal component analysis (PCA) showing distinctive transcriptomic features between resistant cell subline and parental cells. B) Volcano plot showing upregulated (green) and downregulated (red) genes in gemcitabine-resistant cells compared to parental cells. Differentially expressed gene group was determined with p-value < 0.01 and fold-change ≥ 3. C) Venn diagram showing the comparative numerical analysis of DEGs between NOZ GemR and TGBC1 GemR cells. D) GO/pathway terms specific to differentially expressed genes (DEGs) in NOZ GemR (left) and TGBC1 GemR (right). Shared GO terms between GemR cells are indicated in the bar graph (green arrow). The bars represent the percentage of genes associated with the terms. Bar colors represent different functional networks.

Table S6. Differential gene expression in NOZ GemR relative to NOZ parental cells.

**Gene ID**	**Microarray**			**qRT-PCR**		
	**Fold-change**	**Regulation**	***p*-value**	**Fold-change**	**Regulation**	***p*-value**
ABCB1	1.03	Up	0.7455	-0.458	Down	0.2536
ABCC1	-1.94	Down	0.005	-0.599	Down	0.0117
ABCC2	-1.06	Down	0.3044	-0.257	Down	0.3559
ABCC3	1.3	Up	0.6105	-0.469	Down	0.0137
ABCG2	-1.14	Down	0.0583	-0.930	Down	0.0505
SLC29A1	-1.97	Down	0.0085	-2.230	Down	0.0008
SLC29A2	-2.01	Down	0.0197	-2.013	Down	0.0002
CDA	19.61	Up	0.0033	2.959	Up	0.0007
SNAI1	-1.1	Down	0.2526	-1.185	Down	0.0105
SNAI2	3.76	Up	0.0066	2.451	Up	0.0073
ZEB1	1.14	Up	0.4243	0.713	Up	0.0017
VIM	1.42	Up	0.0106	2.213	Up	0.0020
CDH1	-1.12	Down	0.0223	-2.443	Down	0.0013
CDH2	1.16	Up	0.0315	3.151	Up	0.0211

Table S7. Differential gene expression in TGBC1 GemR relative to TGBC1 parental cells.

**Gene ID**	**Microarray**			**qRT-PCR**		
	**Fold-change**	**Regulation**	***p*-value**	**Fold-change**	**Regulation**	***p*-value**
ABCB1	1.28	Up	0.2116	2.20	Up	0.3333
ABCC1	-1.03	Down	0.6151	0.48	Up	0.2653
ABCC2	7.12	Up	0.0356	3.02	Up	0.0308
ABCC3	-1.16	Down	0.4380	2.00	Up	0.3333
ABCG2	1.73	Up	0.2997	-1.00	Down	0.100
SLC29A1	-1.33	Down	0.0361	-0.97	Down	0.0745
SLC29A2	-6.19	Down	0.0151	-5.30	Down	0.0004
CDA	2.32	Up	0.0091	1.33	Up	0.0039
SNAI1	-1.52	Down	0.2893	0.90	Up	0.0656
SNAI2	2.49	Up	0.0003	1.71	Up	0.0176
ZEB1	3.85	Up	0.0005	2.72	Up	0.0013
VIM	48.10	Up	1.57e-05	7.94	Up	0.0264
CDH1	-13.18	Down	0.0125	-2.78	Down	0.0135
CDH2	-1.20	Down	0.5141	-1.36	Down	0.0083

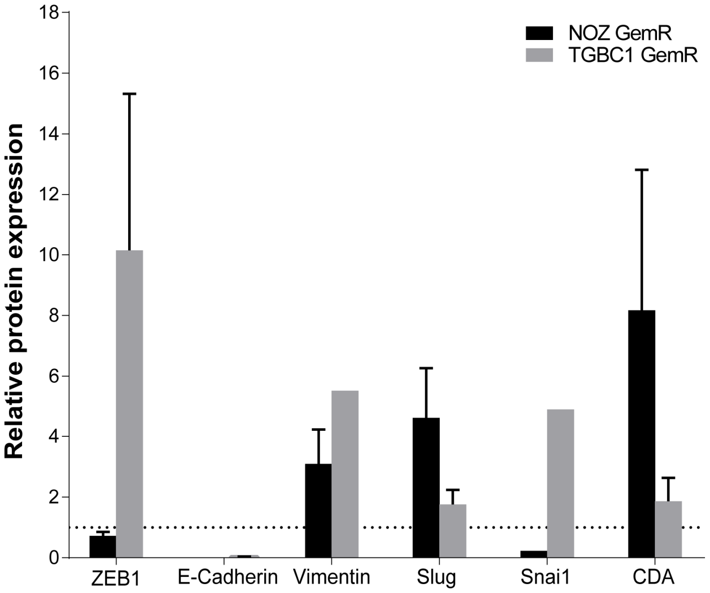

Figure S3. Densitometric analysis based on three independent Western blot experiments (mean ± SD), except for Snai1 (NOZ and TGBC1 GemR) and Vimentin (TGBC1 GemR) where the data from one experiment is shown*. Bands were normalized to beta-actin expression and graph bars represent relative expression to each parental cell line (number of normalized pixels). * *In two independent experiments Vimentin expression was undetectable in parental TGBC1 cells, so it was not possible to determine relative expression. In two experiments, expression of Snai1 could not be determined due to technical issues.*

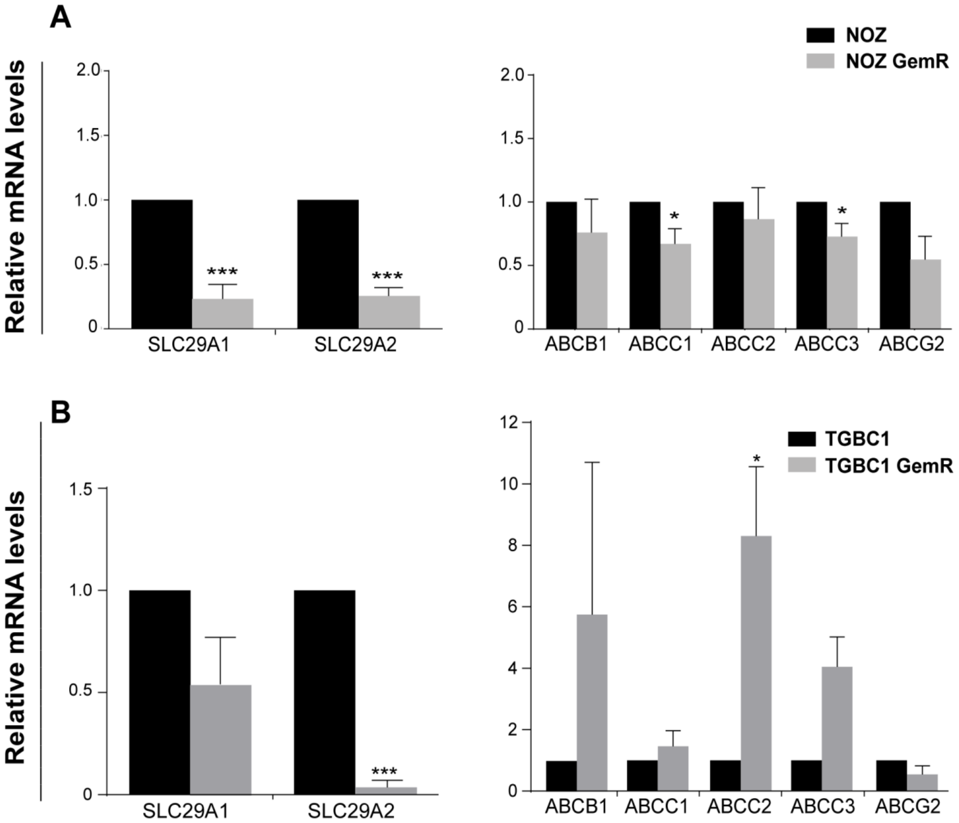

Figure S4. Gemcitabine-resistant cells show dysregulated gene expression of uptake and efflux drug transporters. (A) NOZ GemR cells relative to NOZ cells exhibit downregulation of *SLC29A1* and *SLC29A2,* and slightly decreased transcriptional levels of ABC transporters. (B) TGBC1 GemR display decreased mRNA levels of *SLC29A1* and

SLC29A2, but an increase in *ABCB1*, *ABCC2* and *ABCC3* genes. The RT-qPCR data are presented as relative gene expression ($2^{-\Delta\Delta Ct}$) normalized to *TFCP2* and *QARS* as endogenous reference genes. The relative expression showed for each resistant subline is compared to their respective parental cells (mean ± SD). All data are representative of at least three independent experiments (*$p < 0.05$, ***$p < 0.001$ by a two-tailed Student's t-test with Welch's correction). Graph bars without an asterisk represent results without statistical significance.

Table S8. Relative IC_{50} values of gemcitabine in gallbladder cancer cell lines.

GBC Cell Line	IC_{50} (nM)
NOZ	6.2
G-415	17.5
TGBC1	40.2
TGBC2	90.8

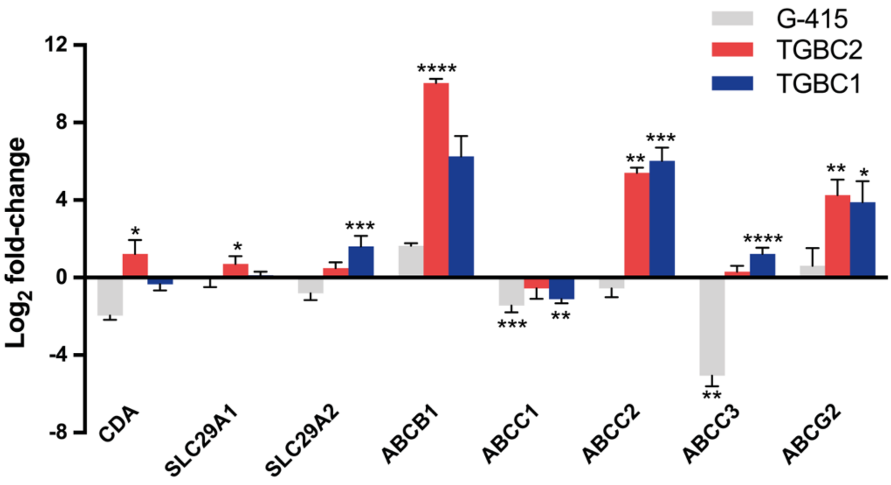

Figure S5. Log_2 fold-change values determined by qPCR for cytidine deaminase (CDA), drug-influx (*SLC29A1* and *SLC29A2*) and drug-efflux transporters (ABCs). * Denotes relative expression values that were significantly different from NOZ (*$p < 0.05$; **$p < 0.01$; ***$p < 0.001$; ****$p < 0.0001$ by one-way ANOVA or Kruskal-Wallis test).

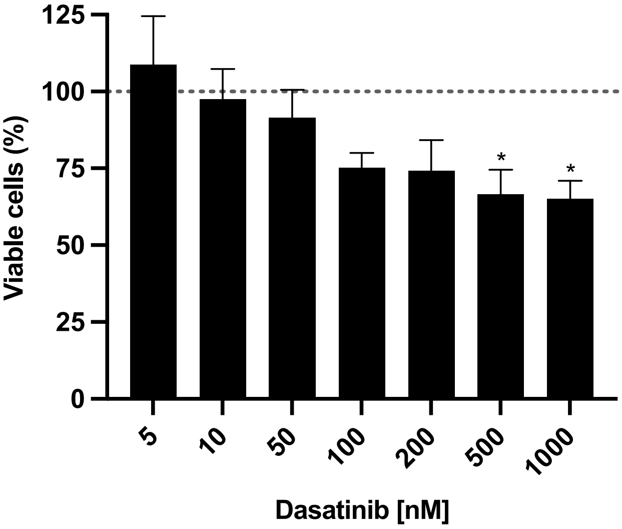

Figure S6. Cell viability of NOZ GemR cells 24 hours after dasatinib treatment. Data (means ± SD) are expressed as percentages of the vehicle control (defined as 100 %). (* $p < 0.05$; Kruskal–Wallis with post hoc Dunn's multiple comparison test).

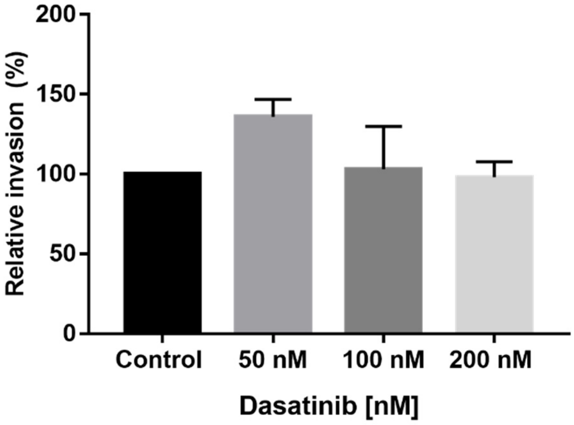

Figure S7. Relative cell invasion in NOZ GemR cells after treatment with dasatinib. All data are representative of three independent experiments with three technical replicates (mean ± SD, analyzed by Kruskall-Wallis test, non-significant differences were observed).

Table S15. List of primers used in the present study for real-time RT-PCR.

Primer	Sequence Forward (5′-3′)	Sequence Reverse (5′-3′)
ABCB1	TGC CCT TGT TAG ACA GCC TCA T	ACA GGC GGT GAG CAA TCA CAA T
ABCC1	TTT TGG CAG CCA GAC TTC TGG A	ACA AAA CTG CAG CCC CAA GGA A
ABCC2	GCC AGC CTG CAA CTT GGG TTA T	AGT GGG CGA ACT CGT TTT GGA T
ABCC3	CTG GAG TCG CTT TCA TGG TCT T	TGC CGT TCA GGA TCT CAC TCA T
ABCG2	TTG GCT GAG GGT TTG GAA CTG T	ACG TGA CCT CCC AGA GCT AGA A
SLC29A1	TCT CCA CTC TTG GCT CTG AC	GGG ACA TGG AGA GAA CAC AG
SLC29A2	AAA GTG ACT GGT CCG TGG TA	GGT CAG AAG GAG AAA CAG CA
CDA	TAC AGG GAC TGG GCA AAG ATG A	GTT CTA AGT CCC AAG GCA GGT T
SNAI1	AGG CTC GAA AGG CCT TCA ACT	TGT GGC TTC GGA TGT GCA TCT
SNAI2	CTC CAT TCC ACG CCC AGC TAC	AGC CAC TGT GGT CCT TGG AG
ZEB1	TCA GCT CCT GCA CTT CTG TCA T	ACC CTG TTA GGC AGT GAG GAA T
VIM	GCC CTT GAC ATT GAG ATT GCC A	TCA ACC AGA GGG AGT GAA TCC A
CDH1	ATC CCC AAG TGC CTG CTT TTG A	CCC CTT TAG GGC CAC ATT TTC T
CDH2	GCC ACG GTT CAA GAA ACT TGC T	GCC CAA ATT GGT TTG CAG CCT A
QARS	ACC TGA ACC TGG CAT CAC TAC A	CCA AGA CGC TCA AAC TGG AAC T
TFCP2	CCC TTG CCA GAT CAG CCA GAT TT	ACG CCG CAC TCC TAC TTC AGT AT

Supplementary methods/Western blot analysis

Cells were washed three times with cold 1X phosphate-buffered saline (PBS) and lysed with RIPA buffer (Thermo Scientific, Rockford, IL, USA) supplemented with protease/phosphatase inhibitors (Thermo Scientific, Rockford, IL, USA). Total proteins were quantified using the BCA protein assay reagent (Pierce™ BCA Protein Assay Kit, Thermo Scientific, Rockford, IL, USA) according to the manufacturer's instructions. Cell extracts containing 30 ug of total protein were subjected to SDS-PAGE using 4-20% Mini-Protean TGX pre-cast gels (Bio-Rad Laboratories, Hercules, CA, USA), and the resolved proteins were transferred to PVDF membranes (Thermo Scientific, Rockford, IL, USA) using the Mini Trans-Blot electrophoretic transfer cell (Bio-Rad Laboratories). After blocking with 1X TBS, 0.1% Tween-20 with 5% w/v nonfat dry milk for 1 h at room temperature, membranes were incubated with the primary antibody diluted in 1X TBS, 0.1% Tween-20 with 5% w/v BSA overnight at 4 °C, with agitation. Next day, blots were incubated in the secondary antibody for 1 h with gentle shaking. Washing steps with 1X TBS, 0.1% Tween-20 were done after both primary and secondary antibody incubations. Antibody-bound protein bands were detected with enhanced chemiluminescence reagent SuperSignal West Dura Substrate (Thermo Scientific, Rockford, IL, USA) and images were acquired using the ImageQuant LAS 500 system (GE Healthcare Life Sciences, Chicago, IL, USA). The quantification of different specific bands was calculated by densitometry using ImageJ v1.5 (National Institute of Health, Bethesda MD, USA). The results were normalized to beta-actin expression and shown as relative expression to each parental cell line.

Supplementary methods/SILAC protocol

Peptides were prepared by an in-solution tryptic digestion protocol with modifications (Rush et al., 2005; Zhong et al., 2012). Briefly, after washed with serum-free DMEM thrice, NOZ cells or NOZ GemR cells were lysed in lysis buffer (20 mM HEPES pH 8.0, 9 M urea, 1 mM sodium orthovanadate, 2.5 mM sodium pyrophosphate, 1 mM glycerophosphate, 10 mM sodium fluoride), sonicated, and cleared by centrifugation at 20,000 × g at 15 °C for 20 min. Equal amounts of protein from NOZ cells and NOZ GemR cells were mixed, reduced with dithiothreitol, and alkylated with iodoacetamide. Protein extracts were diluted in 20 mM HEPES pH 8.0 to a final concentration of 2 M urea and incubated with TPCK-treated trypsin at 25 °C overnight. Protein digests were acidified by 1% trifluoroacetic acid (TFA) and subjected to centrifugation at 2000 × g at room temperature for 5 min. The supernatant of protein digests (25 mg each) was loaded onto a Sep-Pak C18 column (Waters, Cat#WAT051910) equilibrated with 0.1% TFA. Columns were washed with 12 ml of 0.1% TFA and peptides were eluted in 6ml of 40% acetonitrile (ACN) with 0.1% TFA. Eluted peptides were lyophilized and subjected to phosphopeptide enrichment.

Immunoaffinity purification (IAP) of phosphopeptides was carried out as previously described (Rush et al., 2005; Zhong et al., 2012). Briefly, after lyophilization, 40–60 mg of peptide mixture was dissolved in 1.4 ml of IAP buffer (50

mM MOPS, pH 7.2, 10 mM sodium phosphate, 50 mM NaCl) and subjected to centrifugation at 2000 × g at room temperature for 5 min. Before IAP, anti-phosphotyrosine mouse mAb (P-Tyr-1000) beads (Cell Signaling Technology, Danvers, MA, USA) were washed with IAP buffer twice at 4 °C and the pH of the supernatant containing peptides was adjusted to 7.2 by adding 1 M Tris Base. For IAP, the supernatant was incubated with P-Tyr-100 beads (Cell Signaling Technology, Danvers, MA, USA) at 4 °C for 30 min and the beads were washed three times with IAP buffer and then twice with water. Peptides were eluted twice from beads by incubating the beads with 0.1% TFA at room temperature.

Supplementary methods/Mass spectrometric data analysis

The search parameters included: SILAC 2-state, Arg6/Lys6; a maximum of three SILAC labels per peptide; a maximum of two missed cleavages; fixed modification: carbamidomethylation of cysteine; variable modification: protein N-term acetylation, oxidation of methionine, deamination of asparagine and glutamine, and phosphorylation of serine, threonine and tyrosine. The first search and main search peptide tolerance were set to 20 ppm and 4.5 ppm, respectively. The FTMS MS/MS match tolerance was set to 20 ppm. The maximum number of modifications per peptide was set to 6 and the maximum charge was set at 7. The revert type of the target-decoy analysis was chosen. The peptide-spectrum match (PSM) false discovery rate (FDR), protein FDR and the site decoy fraction were set to 0.01. The minimum peptide length was set to 7. The minimal scores for unmodified and modified peptides were 0 and 40, respectively. The minimal delta score for unmodified and modified peptides were 0 and 6, respectively. The minimum of unique and razor peptides for identification was set to 1.

References

Rush, J., A. Moritz, K.A. Lee, A. Guo, V.L. Goss, E.J. Spek, H. Zhang, X.M. Zha, R.D. Polakiewicz and M.J. Comb, Immunoaffinity profiling of tyrosine phosphorylation in cancer cells, Nat Biotechnol 23 (2005), pp. 94-101. https://doi.org/10.1038/nbt1046

Zhong, J., M.S. Kim, R. Chaerkady, X. Wu, T.C. Huang, D. Getnet, C.J. Mitchell, S.M. Palapetta, J. Sharma, R.N. O'Meally, R.N. Cole, A. Yoda, A. Moritz, M.M. Loriaux, J. Rush, D.M. Weinstock, J.W. Tyner and A. Pandey, TSLP signaling network revealed by SILAC-based phosphoproteomics, Mol Cell Proteomics 11 (2012), p. M112 017764. https://doi.org/10.1074/mcp.M112.017764
